# Relationship between diet quality scores and the risk of frailty and mortality in adults across a wide age spectrum

**DOI:** 10.1186/s12916-021-01918-5

**Published:** 2021-03-16

**Authors:** Kulapong Jayanama, Olga Theou, Judith Godin, Leah Cahill, Nitin Shivappa, James R. Hébert, Michael D. Wirth, Yong-Moon Park, Teresa T. Fung, Kenneth Rockwood

**Affiliations:** 1 Chakri Naruebodindra Medical Institute, Faculty of Medicine Ramathibodi Hospital, Mahidol University, Samut Prakan, Thailand; 2 Division of Geriatric Medicine, Dalhousie University & Nova Scotia Health, Halifax, Nova Scotia Canada; 3 School of Physiotherapy, Dalhousie University, Halifax, Nova Scotia Canada; 4 Department of Medicine, Dalhousie University, Halifax, Nova Scotia Canada; 5 Department of Nutrition, Harvard T.H. Chan School of Public Health, Boston, MA USA; 6 Cancer Prevention and Control Program, University of South Carolina, Columbia, SC USA; 7 Department of Epidemiology and Biostatistics, Arnold School of Public Health, University of South Carolina, Columbia, SC USA; 8 Department of Nutrition, Connecting Health Innovations LLC, Columbia, SC USA; 9 College of Nursing, University of South Carolina, Columbia, SC USA; 10 Department of Epidemiology, Fay W. Boozman College of Public Health, University of Arkansas for Medical Sciences, Little Rock, AR USA; 11 Department of Nutrition, Simmons University, Boston, MA USA

**Keywords:** Dietary indices, Dietary score, Nutrition, Frailty, Frailty index, Mortality

## Abstract

**Background:**

Beyond intakes of total energy and individual nutrient, eating patterns may influence health, and thereby the risk of adverse outcomes. How different diet measures relate to frailty—a general measure of increased vulnerability to unfavorable health outcomes—and mortality risk, and how this might vary across the life course, is not known. We investigated the associations of five dietary indices (Nutrition Index (NI), the energy-density Dietary Inflammatory Index (E-DII™), Healthy Eating Index-2015 (HEI-2015), Mediterranean Diet Score (MDS), and Dietary Approaches to Stop Hypertension (DASH)) with frailty and mortality.

**Methods:**

We included 15,249 participants aged ≥ 20 years from the 2007–2012 cohorts of the National Health and Nutrition Examination Survey (NHANES)*.* The NI combined 31 nutrition-related deficits. The E-DII is a literature-derived dietary index associated with inflammation. The HEI-2015 assesses adherence to the Dietary Guidelines of Americans. The MDS represents adherence to the traditional Mediterranean diet. DASH combines macronutrients and micronutrients to prevent hypertension. Frailty was evaluated using a 36-item frailty index. Mortality status was ascertained up to December 31, 2015.

**Results:**

Participants’ mean age was 47.2 ± 16.7 years and 51.7% were women. After adjusting for age, sex, race, educational level, marital and employment status, smoking, BMI, and study cohort, higher NI and E-DII scores and lower HEI-2015, MDS, and DASH scores were individually significantly associated with frailty. All dietary scores were significantly associated with 8-year mortality risk after adjusting for basic covariates and frailty: NI (hazard ratio per 0.1 point, 1.15, 95%CI 1.10–1.21), E-DII (per 1 point, 1.05, 1.01–1.08), HEI-2015 (per 10 points, 0.93, 0.89–0.97), MDS (per 1 point, 0.94, 0.90–0.97), and DASH (per 1 point, 0.96, 0.93–0.99). The associations of E-DII, HEI-2015, and MDS scores with 8-year mortality risk persisted after additionally adjusting for NI.

**Conclusions:**

NI, E-DII, HEI-2015, MDS, and DASH scores are associated with frailty and 8-year mortality risk in adults across all ages. Nevertheless, their mechanisms and sensitivity to predict health outcomes may differ. Nutrition scores have the potential to include measures of both consumption and laboratory and physical measures of exposure.

**Supplementary Information:**

The online version contains supplementary material available at 10.1186/s12916-021-01918-5.

## Background

Across the lifespan, diet plays an important role in growth, development, and health. Food consumption supplies the energy and essential nutritional substrates required for metabolism and homeostasis. Adequate nutrient intake can decrease the incidence of many diseases and specific deficiencies. However, more subtle imbalances in dietary intake and malnutrition are associated with adverse health outcomes such as metabolic syndrome, type 2 diabetes mellitus, cardiovascular diseases, cancer, infection, cognitive impairment, poor quality of life, disability, and mortality [1–3]. Interestingly, diets that are associated with a lower risk of almost any single disease tend to be associated with lower risk of disease in general, making them “healthy diets” [4].

Despite the convergence of dietary factors associated with lower risk, different diet scores are used to measure different aspects of diet quality. Overall, dietary quality pattern can be more important than individual nutrients in predicting major metabolic and non-communicable diseases [5, 6]. Specifically, the Mediterranean diet, a healthy and balanced dietary pattern focusing heavily on fresh fruits and vegetables, is known to reduce the incidence and mortality rate from major cardiovascular events [4] and cancers [7] and improves cognition in older adults [8]. The Dietary Approaches to Stop Hypertension (DASH), which combines macronutrients and micronutrients, is associated with decreased risk of hypertension and lower cardiovascular-related metabolic diseases [9]. The Healthy Eating Index-2015 (HEI-2015), a diet-quality index, is associated with decreased risk of cancer, cardiovascular disease, and all-cause mortality [10, 11]. The Dietary Inflammatory Index (DII®) and energy-adjusted DII (E-DII™) are literature-derived and population-based scores, which are associated with cancer and a number of other non-communicable chronic diseases [12–17]. More recently, they have also been associated with a higher risk of frailty [18–22].

Frailty, understood as the increased vulnerability to adverse outcomes among people of the same chronological age [23], is related to having higher rates of a variety of diseases and health conditions and is common across the adult-aged spectrum [24, 25]. Frailty and malnutrition appear to be reciprocally related; higher levels of frailty are associated with malnutrition [26] and malnutrition is related to higher levels of frailty [27]. Given how common frailty is, and how many illnesses themselves are associated with frailty, there is great interest in whether healthy diets can mitigate frailty and its risks. Previously, a multidimensional intervention that included nutrition management was shown to ameliorate frailty [28]. Whether this effect is specific to the intervention and whether particular dietary components drive this effect are unknown. Seeking to understand the relationship between nutrition and frailty better, we developed a Nutrition Index (based on an accumulation of deficits approach) and demonstrated both that it was associated with frailty and that the two together were independently associated with mortality [27]. Similar to other dietary indices, the Nutrition Index consists of nutrients related to health outcomes. In general, poor nutritional status is reflected in some combination of inappropriate intake, disproportionate body composition, and abnormal blood levels. Therefore, the Nutrition Index also includes nutrition-related blood tests and anthropometric measurements.

Although malnutrition is a major marker of frailty, few studies have examined the association of dietary patterns with frailty in older people [18, 29, 30]. Fewer still have evaluated how multiple dietary scores relate to mortality independent of frailty, and across the adult age spectrum. Therefore, the aims of this study are to (1) assess the association between dietary scores and frailty in an adult population unrestricted by age and (2) to explore the impact of these dietary scores on mortality risk after adjusting for the degree of frailty.

## Methods

### Study population and design

This observational study included 17,713 participants aged 20 years or older from the 2007–2008, 2009–2010, and 2011–2012 cohorts of the National Health and Nutrition Examination Survey (NHANES)*.* NHANES is administered by the Centers for Disease Control and Prevention (CDC) and the National Center for Health Statistics (NCHS) and comprises publicly available 2-year cross-sectional surveys that focus on the health and nutrition of non-institutionalized US residents [31, 32]. Among the 15,287 participants with dietary data, those with missing frailty index scores (*N* = 15) and mortality (*N* = 23) data were excluded from analysis, leaving 15,249 participants with evaluable data.

Each participant provided written informed consent. The NHANES protocol was approved by the institutional review board of the CDC. As a matter of policy, our local Research Ethics Committee does not review secondary analyses of duly approved, publicly available data.

### Dietary scores

Dietary information was assessed using data recalled from the 24 hours (h) prior to the interview. If there were two 24-h recalls available, the first 24-h recall of the providing dietary data was selected for this analysis. Nutrient values derived from the first 24-h recall came from the United States Department of Agriculture (USDA) food composition database. Data from the NHANES dietary interview and the Food Patterns Equivalents Database files were used to calculate the dietary scores. The variables used to calculate the dietary scores are presented in Additional file 1: Table S1.

#### Nutrition Index (NI)

We previously constructed a deficit accumulation Nutrition Index based from 41 nutrition-related parameters included in the 2003–2006 NHANES cohorts [27, 33]. The Nutrition Index used here equally weighted 31 of these nutrition-related parameters measured in the 2007–2012 NHANES: 18 nutrients (energy, energy per weight, protein, protein per weight, carbohydrate, percentage of saturated fat, vitamins A, C, B1, B2, B3, and B6, folate, phosphorous, copper, sodium, selenium, fish oil), 3 anthropometric measurements (body mass index, body weight change in the past year, waist circumference), and 10 nutrition-related blood tests (lymphocyte count, hemoglobin, mean corpuscular volume and serum albumin, vitamin D, iron, creatinine, triglyceride, high density lipoprotein (HDL)-cholesterol, and glucose). The Nutrition Index score counts the number of nutritional deficits in an individual in relation to the total deficits considered, therefore yielding values that range between 0 and 1. A higher score represents worse nutritional status. For a sensitivity analysis, we also split the Nutrition Index into two indices: the Nutrition Index-nutrient, which included only the 18 nutrient items, and the Nutrition Index-lab/exam, which included the 3 anthropometric measurements and the 10 nutrition-related blood tests.

#### Energy-density Dietary Inflammatory Index (E-DII™)

The E-DII, which is derived using procedures similar to the DII, is a literature-derived dietary index developed by reviewing and scoring 1943 peer reviewed articles that examined the association between 45 dietary parameters and inflammation which were used to derive “inflammatory effect scores”. These were then standardized against a global database of intake for the 45 dietary parameters. Full details on the scoring can be found elsewhere [34]. Procedures for computing the E-DII are identical to those used for DII computation except the reference database is itself energy adjusted so that each parameter is expressed per thousand kilocalories (1000 kcal) [35, 36]. The parameters available for E-DII computation in this study are protein; carbohydrate; total fat; saturated fat; monounsaturated fat; polyunsaturated fat; omega-3 fatty acids; omega-6 fatty acids; cholesterol; fiber; vitamins A, B1, B2, B3, B6, B12, C, D, and E; folate; beta carotene; iron; magnesium; selenium; zinc; caffeine; and alcohol. A higher score indicates a more pro-inflammatory dietary intake [37].

#### Healthy Eating Index-2015 (HEI-2015)

The HEI-2015 is a diet quality index developed by the USDA’s Center for Nutrition Policy and Promotion to assess adherence to the Dietary Guidelines of Americans (DGA) [38]. HEI-2015 has been developed from the HEI-2010 by replacing empty calories with saturated fat and added sugar and focuses on the consumption of total fruits, whole fruits, total vegetables, greens and beans, whole grains, dairy foods, total protein foods, seafood and plant proteins, fatty acids, refined grains, sodium, added sugars, and saturated fats [11]. The score ranges between 0 and 100 points. A higher score reflects healthier eating.

#### Mediterranean Diet Score (MDS)

The Mediterranean Diet Score (MDS) assesses adherence to the traditional Mediterranean diet, composed of an abundant consumption of fruits, vegetables, nuts and whole grains, moderate to high amounts of fish and dairy products, low amounts of red meat, consumption of olive oil as the main source of fat, and mild to moderate consumption of wine [39, 40]. This dietary score includes 10 components: one point score for equal or higher than median intake of non-refined cereals, legumes, fruits and nuts, vegetables (excluding potatoes), fish (shrimp, clams, and fish), and ratio of monounsaturated fatty acids to saturated fatty acids; one point score for lower than median intake of red meat and products, poultry, and dairy products; and one point score for alcohol consumption (14–28 g/day in female; 28–70 g/day in male) [41, 42]. Potatoes were excluded in the calculation for NHANES due to differences in preparation methods between the USA and Europe [43]. MDS score ranges between 0 and 10 points and a higher score indicates the better adherence to Mediterranean diet pattern.

#### Dietary Approaches to Stop Hypertension (DASH)

The DASH dietary pattern focuses on high amounts of fruits, vegetables, and low-fat dairy products, aimed at lowering blood pressure [44]. Here, we calculated a DASH score based on the nine-item, nutrient-based DASH index: protein, fiber, magnesium, calcium, potassium, total fat, saturated fat, cholesterol, and sodium. Meeting the goal for each component provides one point, meeting an intermediate goal between the DASH diet goal and the nutrient content of the DASH control diet provides 0.5 points, and meeting neither goal gives zero points. The optimal micronutrient targets are energy adjusted per 1000 kcal. The control diet targets are from the previous DASH study [45, 46]. This pattern score ranges between 0 and 9 points, where higher scores indicate greater adherence to the DASH dietary pattern.

### Frailty index

Frailty was evaluated using a 36-item deficit accumulation frailty index modified from previous NHANES studies [25, 27], including self-report health, vital signs, and laboratory tests (Additional file 1: Table S2). The frailty index was calculated by counting the number of individual deficits and dividing by the total number of possible deficits. No items related to dietary intake or nutritional status were included in this frailty index. Scores ranged between 0 and 1, where a higher score is indicative of higher frailty.

### Mortality

Mortality status was identified from the death certificate records in the National Death Index through December 31, 2015 [47]. Survival time was counted from the date of the clinical examination (2007–2012); all participants had between 3 and 8 years of follow-up. We examined mortality rate until 3 years and until 8 years, and time to mortality up to 3 years and up to 8 years.

### Statistical analysis

Participants’ characteristics, as a whole and stratified by mortality status, were presented as mean ± standard deviation (SD) for continuous variables or as frequency (%) for categorical or ordinal variables. All percentages and means were weighted using the 6-year sampling weights constructed from the sampling weights provided by NHANES for the general US population-based estimates. The correlation between dietary scores was tested using Pearson’s correlation (*r*). If the correlation between dietary scores was not strong, the pairs of dietary scores could be included in the same model. Regarding objective 1, the association between each dietary score and frailty was analyzed using ordinary least squares (OLS) regression models and we present unstandardized beta-coefficients with 95% confidence intervals (CI) and standardized beta-coefficients. For objective 2, the mortality risk of each dietary score was assessed using Cox regression models, and we present hazard ratios (HR) with the associated 95% CIs and cumulative survival probability curve. Time to death was tested for both up to 3 years and up to 8 years of follow-up. In both the OLS and Cox regressions, linear and non-linear (squared and cubic) associations were examined. For non-linear associations, the model included the dietary score and the square of the dietary score (allowing a line with one bend), and the cubic model included the dietary score, the square of the dietary score, and the cube of the dietary score (allowing a line with two bends). All regression models were adjusted for potential confounders (provided by NHANES), as basic covariates, including age (continuous, in years), sex (male and female), race (non-Hispanic white, non-Hispanic black, Hispanic, and others), education level (less than high school, high school, some college/associated education, and college graduate or higher), marital status (married, widowed, divorced or separated, and never married), employment status (working and non-working), smoking (never, former, and current), body mass index (BMI) (< 18.5 kg/m^2^, 18.5–24.9, 25.0–29.9, and ≥ 30.0), and study cohort (number). Annual household income was not included as a covariate due to a high rate of missing data (average 9.1%). Energy intake was included or used for adjusting when creating all dietary scores except MDS. Therefore, energy intake (continuous score in Kcal) was added as a covariate in the regression models where MDS was a single dietary score but not when that model was additionally adjusted for Nutrition Index (Nutrition Index was also adjusted for energy intake). The Cox regression analyses were adjusted for all factors listed above and frailty index (continuous score). To control for the overall nutrition-related accumulation deficits in the association of dietary scores with frailty and mortality we also added the Nutrition Index (continuous score) to our regression models. We also compared all other pairs of dietary scores by running regression models with each combination of two dietary scores to assess which ones more often remained independently associated with the outcome (multicollinearity was tested). The effect of age and sex on the association between each dietary score and frailty and the effect of age, sex, and frailty on the association between each dietary score and mortality were examined using an interaction term, in multivariate OLS and Cox regression analyses, respectively. Statistical significance was considered as *p* < 0.05 and all reported probability tests were two-tailed. Statistical analyses were performed using IBM SPSS Statistics for Windows, Version 25.0. Armonk, NY: IBM Corp.

## Results

**Fig. 1:**
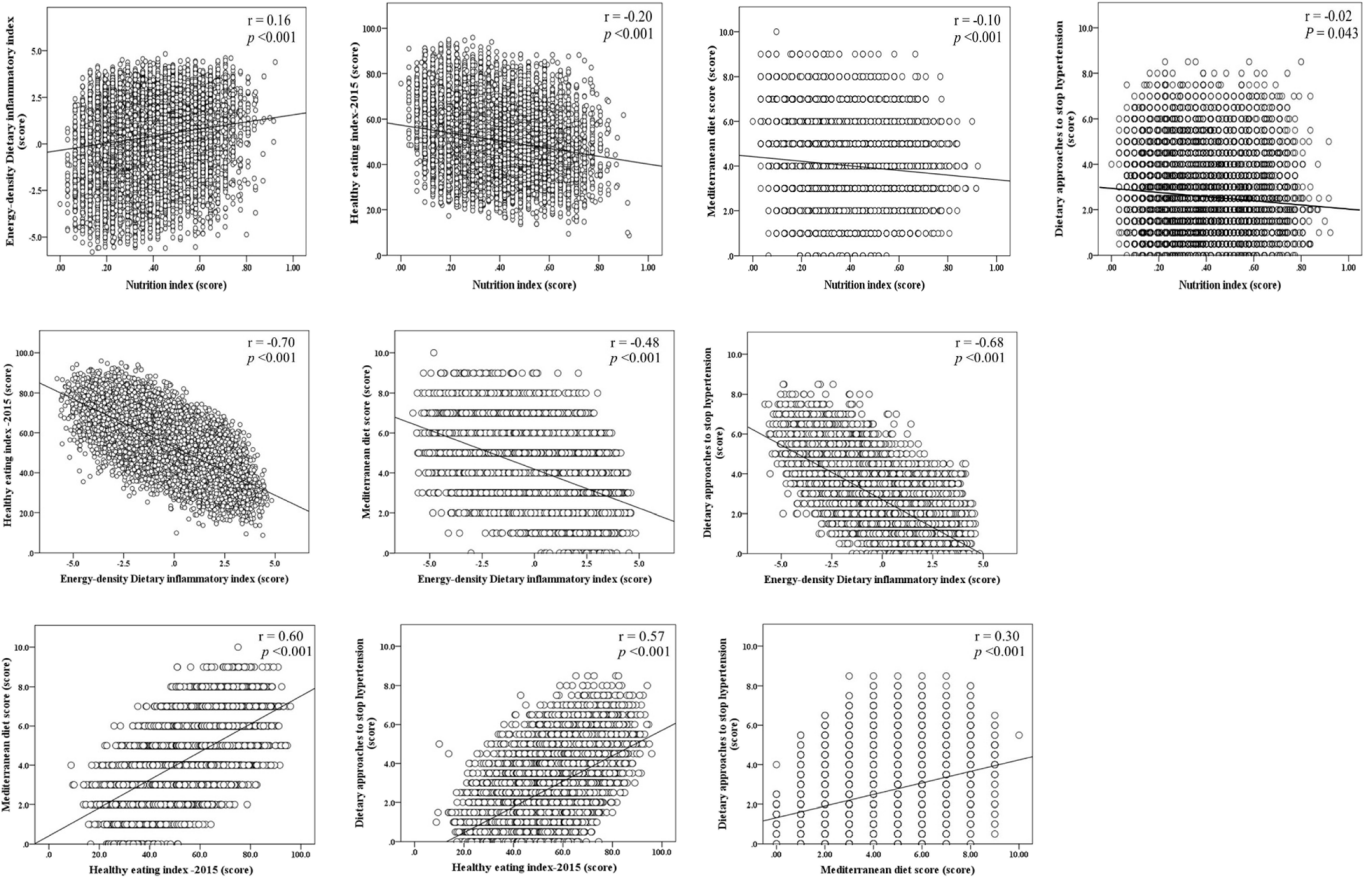
Bivariate scatter plots and linear regression lines between each pair of the dietary scores. Higher NI and E-DII scores and lower HEI-2015, MDS, and DASH scores represent worse dietary pattern/intake

We analyzed 15,249 participants with complete data, of whom 51.7% were female. The mean age was 47.2 ± 16.7 years and mortality rate up to 8 years was 5.3% (*N* = 1171) (Table 1). Older age, female sex, lower energy intake, lower education, non-full-time work, smoking status (former and current), and BMI < 18.5 or ≥ 25 kg/m^2^ were significantly associated with higher frailty (Additional file 1: Table S3). Moderate negative correlations were found between E-DII and HEI-2015 (*r* = − 0.70), and E-DII and DASH (*r* = − 0.68) whereas moderate positive correlations were found between HEI-2015 and MDS (*r* = 0.60), and HEI-2015 and DASH (*r* = 0.57). The correlations between Nutrition Index and the other dietary scores were weak (Fig. 1).

**Table 1:** Descriptive baseline characteristics of participants

Characteristics, mean ± SD or *N* (%)	All participants	8-year mortality	
		Alive ***N*** = 14,078	Deceased ***N*** = 1171
Age (year)	47.2 ± 16.7	46.1 ± 16.1	67.1 ± 14.7
Sex, female	7769 (51.7)	7289 (52.1)	480 (44.9)
Race			
Non-Hispanic white	6949 (69.2)	6263 (68.9)	686 (9.3)
Non-Hispanic black	3142 (10.8)	2907 (10.7)	235 (75.2)
Hispanic	3964 (13.6)	3763 (13.8)	201 (11.3)
Other	1194 (6.4)	1145 (6.5)	49 (4.2)
Education			
Less than high school	4170 (18.2)	3702 (17.4)	468 (32.4)
High school	3480 (22.6)	3189 (22.4)	291 (26.9)
Some college/ associate education	4291 (30.7)	4031 (31.0)	260 (25.8)
College graduate or more	3291 (28.5)	3143 (29.2)	148 (15.0)
Marital status			
Married	9020 (63.5)	8444 (64.2)	576 (50.7)
Widowed	1276 (5.7)	946 (4.6)	330 (26.5)
Divorced or separated	2179 (12.6)	2002 (12.5)	177 (14.5)
Never married	2769 (18.1)	2681 (18.7)	88 (8.3)
Full-time working	8278 (63.0)	8069 (65.3)	209 (22.7)
Smoking status			
Never	8253 (54.6)	7771 (55.3)	482 (42.1)
Former	3739 (24.7)	3268 (23.9)	471 (38.7)
Current	3252 (20.7)	3034 (20.8)	218 (19.2)
Body mass index (kg/m^2^)			
< 18.5	251 (1.7)	221 (1.6)	30 (2.9)
18.5–24.9	4174 (29.5)	3864 (29.6)	310 (27.8)
25.0–29.9	5071 (33.9)	4668 (33.8)	403 (34.7)
≥ 30	5595 (34.9)	5224 (35.0)	371 (34.6)
Energy intake (Kcal)	2182.3 ± 999.1	2199.1 ± 1000.6	1882.7 ± 920.8
NI score (0 to 1)	0.34 ± 0.15	0.33 ± 0.15	0.41 ± 0.16
E-DII score (− 5.81 to 4.82)	0.35 ± 1.97	0.37 ± 1.97	0.15 ± 1.92
HEI-2015 score (0 to 100)	51.2 ± 13.6	51.1 ± 13.6	51.8 ± 13.1
MDS (0 to 10)	4.0 ± 1.6	4.0 ± 1.6	4.0 ± 1.5
DASH score (0 to 9)	2.5 ± 1.6	2.5 ± 1.5	2.5 ± 1.6
FI score (0 to 1)	0.10 ± 0.10	0.09 ± 0.09	0.24 ± 0.15

*DASH* Dietary Approaches to Stop Hypertension, *E-DII* Energy-density Dietary Inflammatory Index, *FI* Frailty index, *HEI-2015* Healthy Eating Index-2015, *Kcal* kilocalories, *kg* kilogram, *m* meter, *MDS* Mediterranean Diet Score, *NI* Nutrition Index. Higher NI and E-DII scores and lower HEI-2015, MDS, and DASH scores represent worse dietary pattern/intake. The percentages and mean values are weighted with sampling weights

**Fig. 2:**
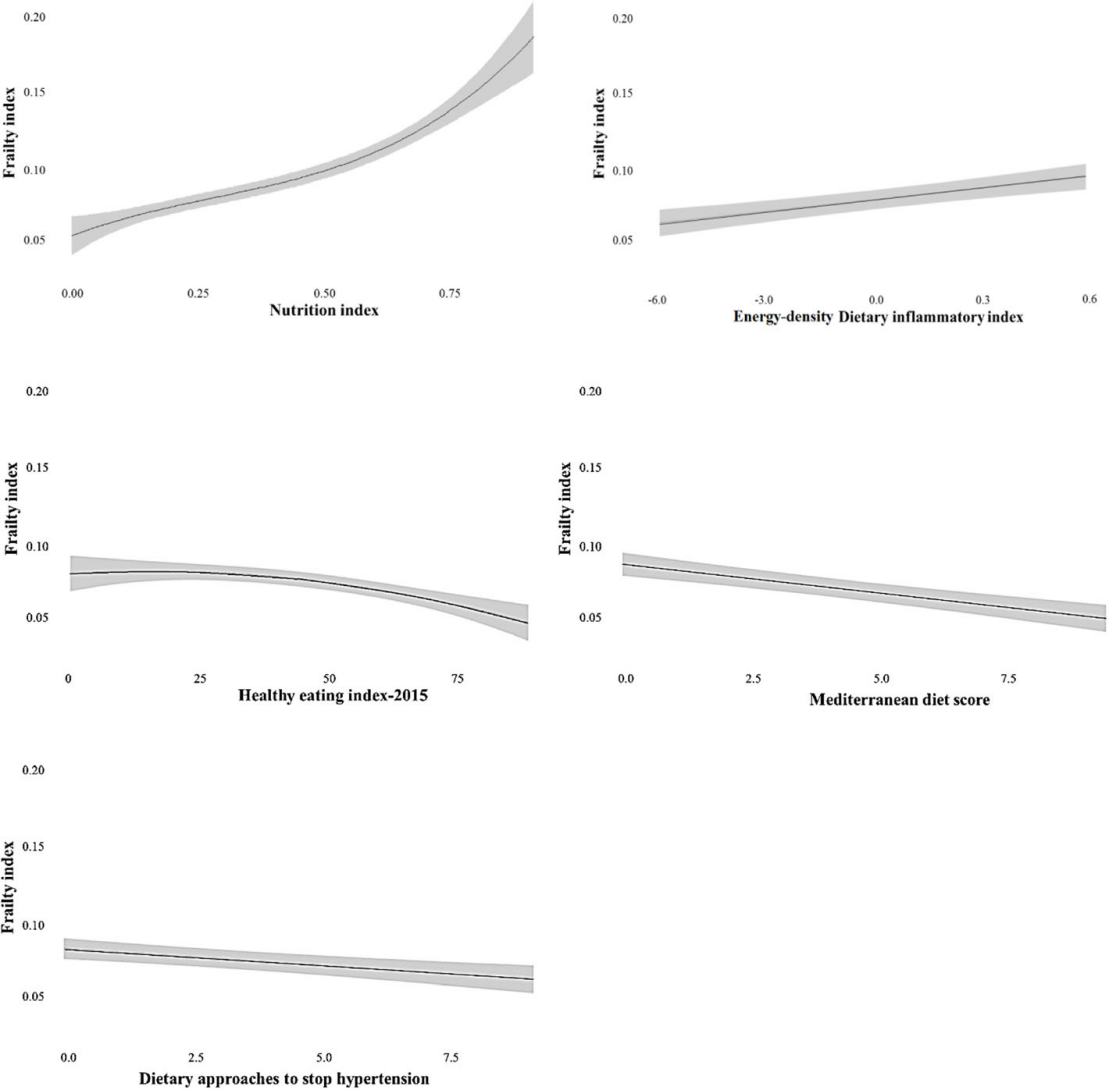
Relationship between dietary scores and frailty. Here we present frailty index predictive values with 95% confidence interval for each level of dietary scores. Regression models were adjusted for basic covariates (age, sex, race, education level, marital status, employment status, smoking, study cohort, and body mass index). Higher Nutrition Index and Energy-density Dietary Inflammatory Index scores and lower Healthy Eating Index-2015, Mediterranean Diet Score, and Dietary Approaches to Stop Hypertension scores represent worse dietary pattern/intake

Regarding objective 1 (to assess the association between dietary scores and frailty), higher Nutrition Index and E-DII scores and lower HEI-2015, MDS, and DASH scores were significantly associated with higher frailty (Table 2 and Fig. 2). The HEI-2015, MDS, and DASH were also associated with frailty in the same direction after controlling for the Nutrition Index; Nutrition Index remained a significant predictor.

**Table 2:** Relationship between dietary scores and frailty, using multivariable-adjusted ordinary least squares regression analyses

Dietary scores	Unstandardized beta-coefficients (95%CI)	Standardized beta-coefficients	***p*** value
NI (per 0.1 point)	0.014 (0.002 to 0.025)	0.19	0.017
NI squared	− 0.003 (− 0.006 to 0.000)	− 0.32	0.061
NI cubic	0.000 (0.000 to 0.001)	0.26	0.008
E-DII (per 1 point)	0.002 (0.001 to 0.002)	0.03	< 0.001
HEI-2015 (per 10 points)	0.003 (− 0.003 to 0.010)	0.04	0.307
HEI-2015 squared	− 0.001 (− 0.001 to 0.000)	− 0.09	0.022
MDS (per 1 point)*	− 0.003 (− 0.004 to − 0.002)	− 0.05	< 0.001
DASH (per 1 point)	− 0.001 (− 0.002 to − 0.001)	− 0.02	0.003
E-DII (per 1 point)	0.000 (0.000 to 0.001)	0.01	0.293
NI (per 0.1 point)	0.008 (0.007 to 0.009)	0.12	< 0.001
HEI-2015 (per 10 points)	0.006 (0.000 to 0.012)	0.08	0.048
HEI-2015 squared	− 0.001 (−0.001 to 0.000)	− 0.10	0.008
NI (per 0.1 point)	0.008 (0.007 to 0.009)	0.11	< 0.001
MDS (per 1 point)	− 0.002 (− 0.003 to − 0.001)	− 0.03	< 0.001
NI (per 0.1 point)	0.011 (0.010 to 0.012)	0.11	< 0.001
DASH (per 1 point)	− 0.001 (− 0.002 to − 0.001)	− 0.02	0.002
NI (per 0.1 point)	0.018 (0.007 to 0.019)	0.12	< 0.001
HEI-2015 (per 10 points)	− 0.004 (− 0.006 to − 0.003)	− 0.05	< 0.001
E-DII (per 1 point)	0.000 (0.000 to 0.001)	− 0.01	0.427
MDS (per 1 point)	− 0.003 (− 0.004 to − 0.002)	− 0.04	< 0.001
E-DII (per 1 point)	0.000 (0.000 to 0.001)	0.01	0.190
DASH (per 1 point)	0.000 (− 0.001 to 0.001)	0.00	0.916
E-DII (per 1 point)	0.002 (0.001 to 0.003)	0.03	0.002
MDS (per 1 point)	− 0.002 (− 0.003 to 0.000)	− 0.03	0.001
HEI-2015 (per 10 points)	− 0.003 (− 0.004 to − 0.001)	− 0.03	< 0.001
DASH (per 1 point)	0.001 (0.000 to 0.002)	0.01	0.193
HEI-2015 (per 10 points)	− 0.004 (− 0.006 to − 0.003)	− 0.05	< 0.001
DASH (per 1 point)	0.000 (− 0.001 to 0.000)	− 0.01	0.302
MDS (per 1 point)	− 0.003 (− 0.004 to − 0.002)	− 0.04	< 0.001

Higher NI and E-DII scores and lower HEI-2015, MDS, and DASH scores represent worse dietary pattern/intake. All regression models were adjusted for age, sex, race, educational level, marital status, employment status, smoking, study cohort and BMI. In an initial model we tested the linear relationship, in the second model we added the squared term, and in the third model we added the cubic term. We present results only for the highest order model that was statistically significant. If none of the models were statistically significant, we present the linear model

*BMI* body mass index, *DASH* Dietary Approaches to Stop Hypertension, *E-DII* Energy-density Dietary Inflammatory Index, *FI* Frailty index, *HEI-2015* Healthy Eating Index-2015, *MDS* Mediterranean Diet Score, *NI* Nutrition Index

*This regression model was additionally adjusted for energy intake. The standardized beta-coefficients were calculated by multiplying the unstandardized coefficient by the ratio of the standard deviations of the dietary scores and frailty index

Regarding objective 2 (to assess the impact of these dietary scores on mortality risk after adjusting for the degree of frailty), higher Nutrition Index, lower HEI-2015, and lower MDS scores were associated with 3-year mortality and all dietary scores (higher Nutrition Index and E-DII, and lower HEI-2015, MDS, and DASH scores) were associated with 8-year mortality risk; these results persisted after adjusting for the frailty index. After controlling for the Nutrition Index, none of the scores were associated with 3-year mortality, however the E-DII, HEI-2015 and MDS scores were significantly associated with 8-year mortality. The Nutrition Index remained a significant predictor of 3- and 8-year mortality in all Cox regression models. When we compared the other pair of dietary scores, none of them predicted 3-year mortality independently. HEI-2015 and MDS remained significant predictors of 8-year mortality except for the model where they were both added together, and E-DII remained a significant predictor of 8-year mortality except when it was included in the same model with DASH (Table 3).

**Table 3:** Relationship between dietary scores and mortality, using multivariable-adjusted Cox regression analysis

Dietary scores	3-year mortality Deceased, ***N*** (%) = 509 (2.4)		8-year mortality Deceased, ***N*** (%) = 1171 (5.3)	
	Hazard ratio (95%CI)	***p*** value	Hazard ratio (95%CI)	***p*** value
Adjusted for age, sex, race, educational level, marital status, employment status, smoking, study cohort, and BMI (basic covariates)				
NI (per 0.1 point)	1.16 (1.09,1.22)	< 0.001	1.14 (1.09,1.18)	< 0.001
E-DII (per 1 point)	1.03 (0.98,1.08)	0.201	1.04 (1.01,1.08)	0.008
HEI-2015 (per 10 points)	0.92 (0.86,0.98)	0.015	0.92 (0.88,0.96)	< 0.001
MDS (per 1 point)*	0.93 (0.87,0.98)	0.013	0.93 (0.89,0.96)	< 0.001
DASH (per 1 point)	0.96 (0.90,1.01)	0.121	0.96 (0.93,0.99)	0.038
Adjusted for basic covariates and FI				
NI (per 0.1 point)	1.11 (1.04,1.17)	0.001	1.15 (1.10,1.21)	< 0.001
E-DII (per 1 point)	1.03 (0.99,1.08)	0.185	1.05 (1.01,1.08)	0.007
HEI-2015 (per 10 points)	0.93 (0.86,0.99)	0.028	0.93 (0.89,0.97)	0.002
MDS (per 1 point)*	0.94 (0.88,0.99)	0.046	0.94 (0.90,0.97)	0.001
DASH (per 1 point)	0.96 (0.90,1.01)	0.113	0.96 (0.93,0.99)	0.040
E-DII (per 1 point)	1.01 (0.96,1.06)	0.791	1.02 (0.99,1.06)	0.198
E-DII squared	0.98 (0.96,1.00)	0.072	0.99 (0.97,0.99)	0.043
NI (per 0.1 point)	1.10 (1.04,1.17)	0.002	1.08 (1.04,1.13)	< 0.001
HEI-2015 (per 10 points)	0.95 (0.88,1.02)	0.164	0.95 (0.91,0.99)	0.037
NI (per 0.1 point)	1.09 (1.04,1.16)	0.005	1.08 (1.04,1.12)	< 0.001
MDS (per 1 point)	0.95 (0.89,1.01)	0.106	0.94 (0.91,0.98)	0.004
NI (per 0.1 point)	1.10 (1.04,1.17)	0.002	1.08 (1.04,1.13)	< 0.001
DASH (per 1 point)	0.96 (0.91,1.02)	0.166	0.97 (0.93,1.00)	0.062
NI (per 0.1 point)	1.10 (1.04,1.17)	0.001	1.09 (1.05,1.13)	< 0.001
HEI-2015 (per 10 points)	0.91 (0.83,1.00)	0.057	0.94 (0.88,0.99)	0.045
E-DII (per 1 point)	0.98 (0.92,1.05)	0.595	1.01 (0.97,1.05)	0.724
E-DII squared	0.98 (0.96,1.00)	0.057	0.99 (0.97,0.99)	0.036
MDS (per 1 point)	0.94 (0.88,1.01)	0.083	0.94 (0.90,0.99)	0.009
E-DII (per 1 point)	1.01 (0.95,1.06)	0.855	1.02 (0.98,1.06)	0.309
E-DII squared	0.98 (0.96,1.00)	0.058	0.99 (0.97,0.99)	0.033
DASH (per 1 point)	0.97 (0.90,1.05)	0.411	0.99 (0.94,1.04)	0.735
E-DII (per 1 point)	1.01 (0.95,1.08)	0.689	1.04 (0.99,1.08)	0.090
MDS (per 1 point)	0.97 (0.90,1.04)	0.352	0.96 (0.91,1.00)	0.059
HEI-2015 (per 10 points)	0.95 (0.87,1.03)	0.188	0.96 (0.91,1.01)	0.113
DASH (per 1 point)	0.99 (0.92,1.06)	0.741	0.99 (0.95,1.04)	0.728
HEI-2015 (per 10 points)	0.93 (0.86,1.01)	0.102	0.94 (0.87,0.99)	0.016
DASH (per 1 point)	0.97 (0.91,1.03)	0.290	0.98 (0.94,1.01)	0.193
MDS (per 1 point)	0.95 (0.98,1.01)	0.093	0.94 (0.90,0.98)	0.004

2007–2012 NHANES cohorts were included in the analysis and mortality was identified up to December 2015. Higher NI and E-DII scores and lower HEI-2015, MDS, and DASH scores represents worse dietary pattern/intake. In an initial model we tested the linear relationship, in the second model we added the squared term, and in the third model, we added the cubic term. We present results only for the highest order model that was statistically significant. If none of the models were statistically significant, we present the linear model

*BMI* body mass index, *DASH* Dietary Approaches to Stop Hypertension, *E-DII* Energy-density Dietary Inflammatory Index, *FI* Frailty index, *HEI-2015* Healthy Eating Index-2015, *MDS* Mediterranean Diet Score, *NI* Nutrition Index

*This regression model was additionally adjusted for energy intake

For a sensitivity analysis, we examined each component of Nutrition Index (Nutrition Index-nutrient and Nutrition Index-lab/exam) and found that both of these indices were associated with higher level of frailty after adjusting for basic covariates. Correlations between Nutrition Index-nutrient and Nutrition Index-lab/exam and the other dietary scores were weak (Additional file 1: Table S4). All dietary scores were significantly associated with frailty after controlling for both Nutrition Index-nutrient and Nutrition Index-lab/exam, except E-DII (Additional file 1: Table S5). Both Nutrition indices (nutrient and lab/exam) were associated with 8-year mortality risk and the Nutrition Index-lab/exam was further associated with 3-year mortality risk. An association with 8-year mortality risk was found with the E-DII, HEI-2015, and MDS after controlling for Nutrition Index-nutrient or Nutrition Index-lab/exam but only in MDS after controlling for both Nutrition Index-nutrient and Nutrition Index-lab/exam (Additional file 1: Table S6).

In both the OLS and Cox regression models, there were no interactions of age and sex with the association between each dietary score and frailty (Additional file 1: Table S7), nor any interactions of age, sex, and frailty with the association between each dietary score and mortality (Additional file 1: Table S8).

## Discussion

This observational study examined the relationship of five dietary scores (Nutrition Index, E-DII, HEI-2015, MDS, and DASH) with frailty measured using a frailty index and with mortality after having considered the degree of health deficit accumulation in adults of all ages. We found that each dietary measure (higher Nutrition Index and E-DII scores, and lower HEI-2015, MDS, and DASH scores) was associated with higher frailty risk. When controlling for frailty, adherence to dietary scores was associated with 3-year mortality risk (short-term), but here the result depended more on the measure, being seen only with higher Nutrition Index score, and lower HEI-2015 and MDS scores. The association between dietary scores and greater risk of death was more robust for 8-year (medium-term) mortality, elicited among all measures of nutrition intake, i.e., higher Nutrition Index and E-DII scores, and lower HEI-2015, MDS, and DASH scores. (Table 4).

**Table 4:** Summary of the associations between each dietary score and frailty and between each dietary score and mortality

Dietary scores	Frailty		3-year mortality		8-year mortality	
	Adjusted for covariates	Adjusted for covariates + NI	Adjusted for covariates + FI	Adjusted for covariates + FI + NI	Adjusted for covariates + FI	Adjusted for covariates + FI + NI
NI	Non-linear	N/A	+	N/A	+	N/A
E-DII	+	X	X	X	+	Non-linear
HEI-2015	Non-linear	Non-linear	–	X	–	–
MDS	–	–	–	X	–	–
DASH	–	–	X	X	–	X
NI-nutrient	+	N/A	X	N/A	+	N/A
NI-lab/exam	Non-linear	N/A	+	N/A	+	N/A

+ positive association, − reverse association, X no association, N/A not applicable

2007–2012 NHANES cohorts were included in the analysis and mortality was identified up to December 2015. We separated the 31-item nutrition Index into two indices: the NI-nutrient which included only the 18 nutrients and the NI-lab/exam which included 10 nutrition-related blood tests and 3 anthropometric measurements. Higher NI and E-DII scores and lower HEI-2015, MDS, and DASH scores represents worse dietary pattern/intake. Covariates in regression models and Cox regression models were age, sex, race, educational level, marital status, employment status, smoking, study cohort and BMI. The actual scores can be found in Tables 2 and 3, and Additional file 1: Table S5 and S6

*DASH* Dietary Approaches to Stop Hypertension, *E-DII* Energy-density Dietary Inflammatory Index, *FI* Frailty index, *HEI-2015* Healthy Eating Index-2015, *MDS* Mediterranean Diet Score, *NI* Nutrition Index

Similar to our previous study [27], we found that higher (worse) Nutrition Index score—which measures both intake and nutritional status—was significantly associated with higher frailty and with higher mortality risk independent of frailty. Even controlling for the degree of frailty, the Nutrition Index predicted both short-term and medium-term mortality risk. As health deficits accumulate, poor nutritional status could be a factor of frailty. For this reason, we excluded items related to dietary intake or nutritional status from the frailty index. While the Nutrition Index included serum glucose, triglyceride, and HDL-cholesterol, it did not assess underlying diseases related to metabolic syndrome (e.g., diabetes mellitus, high blood pressure) that were included in the frailty index. Patients diagnosed with diabetes mellitus, appropriate dietary intake, and well-controlled serum glucose could have better nutritional status and lower adverse outcomes comparing those with poor-controlled diet intake and serum glucose. This emphasizes that optimized nutritional interventions could modify the Nutrition Index and therefore influence clinical outcomes.

Of the two components of the Nutrition Index, the Nutrition Index-lab/exam was more strongly associated with mortality than the Nutrition Index-nutrient component, which reflected consumption. Moreover, the Nutrition Index-lab/exam was the strongest predictor of 3-year and 8-year mortality risk. The quantity of dietary intake is important and relates to worsening health outcomes. Inasmuch as blood tests and physical examinations (especially anthropometric measurements) yield estimates with narrower variances, they may allow earlier identification of health deficit accumulations and better detection of abnormal physiological status beyond malnutrition. Therefore, blood tests and physical examinations may be more closely related to frailty and can be a more sensitive predictor of mortality [37, 48]. However, nutrient consumption was based on a single 24-h recall and people could inaccurately remember the amount of a food that they ate. For these reasons, all nutrition-related parameters including nutrients, blood tests, and physical exams should be considered when investigating the relationship between nutrition and health outcomes.

The present study revealed associations of higher E-DII and lower HEI-2015, MDS, and DASH scores with higher frailty risk (using frailty index) and higher 8-year mortality risk when controlling for frailty. Lower HEI-2015 and MDS scores were associated with higher 3-year mortality risk. In addition, we found associations of HEI-2015, MDS, and DASH scores with frailty after controlling for the Nutrition Index. We also found associations of HEI-2015, and MDS with medium-term (8-year) mortality after controlling for the Nutrition Index. These findings support earlier studies [11, 20, 22, 40, 49–53] that reported relationships between these scores and adverse health outcomes including cardiovascular disease, brain health, cancer, frailty phenotype, and mortality. These varied dietary scores have similar relationships with frailty and mortality due to the inclusion of healthy nutrients. Notably, the pathophysiological links of these dietary scores to frailty and mortality are different; higher E-DII is associated with higher inflammatory status [54, 55]; higher HEI-2015 score, representing overall diet quality following USDA and DGA recommendations, and Mediterranean-style diet (MDS) are related to sarcopenia and age-related chronic disease "prevention" [56]; and higher DASH score improves blood pressure, lipid profile, and inflammatory status [57]. The independent association of HEI-2015, MDS consumption, and the Nutrition Index with frailty and mortality may reflect that these dietary scores are individually important and their pathogenesis in frailty and mortality could differ. For example, the association of Nutrition Index score could be related to the current nutritional status (adequate intake and nourishment) and the association of HEI-2015 and MDS could result from the balance and nutrient-specific effect of a healthy diet.

This study examined the publicly available and large population-based NHANES data in which the protocol was rigorous and well-controlled. Dietary data were systematically recorded and calculated. Mortality was extracted from death certificate data and had a long follow-up period. All analyses were controlled for various potential confounding factors. Nonetheless, there are some limitations to be aware of when interpreting these results: (1) NHANES is a cross-sectional design and the causal relationship between frailty and nutrition cannot be evaluated directly because of the inability to evaluate temporal relationships in the data; (2) dietary data were recorded using 24-h recall, so day-to-day variation could not be measured, and food intake could have changed during the study period and therefore recall data may not represent participants’ long-term dietary patterns; (3) the estimates of dietary scores were based on self-reported dietary data, not including supplement use; and (4) reverse causation could be possible, as dietary consumption and nutritional status may be altered when people start to feel unwell. Future randomized controlled trials are required to confirm the effect of the change in each dietary score on frailty and mortality risk.

## Conclusions

Even though adequate energy and protein intake are essential, and some macronutrients and micronutrients are related to frailty and mortality, overall daily dietary intakes—including more than one nutrient as an eating pattern—should be taken into consideration. This study revealed that the Nutrition Index, E-DII, HEI-2015, MDS, and DASH were associated with frailty and mortality risk across the life course. Nevertheless, their mechanisms within health outcomes may differ. Regarding the Nutrition Index, the accumulation of blood test and physical examination deficits may be a more sensitive predictor of adverse health outcomes such as frailty and mortality, compared to the accumulation of deficits solely regarding dietary intake.

## Supplementary Information

**Additional file 1: Table S1.** Dietary variables included in calculations of the dietary scores. **Table S2.** 36-item frailty index. **Table S3.** Association of participants’ baseline characteristics with frailty, using univariate linear regression analyses and with mortality risk, using Cox regression analysis. **Table S4.** Correlation of the Nutrition Index-nutrient and Nutrition Index-lab/exam with other dietary scores. **Table S5.** Relationship of the Nutrition Index-nutrient and Nutrition Index-lab/exam with frailty, using multivariable-adjusted ordinary least squares regression analyses. **Table S6.** Relationship of the Nutrition Index-nutrient and Nutrition Index-lab/exam with mortality, using multivariable-adjusted Cox regression analysis. **Table S7.** Coefficients and *p* values for the 3-way interactions of each dietary score with age and sex on frailty. **Table S8.** Coefficients and *p* values for the 4-way interactions of each dietary score with age, sex, and frailty on mortality.

## Data Availability

The National Health and Nutrition Examination Survey (NHANES) data are publicly available at https://www.cdc.gov/nchs/nhanes/index.htm.

## Abbreviations

BMI: Body mass index
CDC: Centers for Disease Control and Prevention
CI: Confidence interval
DASH: Dietary Approaches to Stop Hypertension
DGA: Dietary Guidelines of Americans
E-DII: Energy-density Dietary Inflammatory Index
HEI-2015: Healthy Eating Index-2015
HR: Hazard ratio
kg: Kilogram
m: Meter
MDS: Mediterranean Diet Score
NCHS: National Center for Health Statistics
NHANES: National Health and Nutrition Examination Survey
OLS: Ordinary least squares
USDA: United States Department of Agriculture
