# Supplementary material for: Relationship between diet quality scores and the risk of frailty and mortality in adults across a wide age spectrum
Source: BMC Med. 2021 Mar 16;19:64. doi: 10.1186/s12916-021-01918-5 (PMC7962372; doi:10.1186/s12916-021-01918-5)
Supplement: Supplementary file 1 — Additional file 1: Table S1. Dietary variables included in calculations of the dietary scores. Table S2. 36-item frailty index. Table S3. Association of participants’ baseline characteristics with frailty, using univariate linear regression analyses and with mortality risk, using Cox regression analysis. Table S4. Correlation of the Nutrition Index-nutrient and Nutrition Index-lab/exam with other dietary scores. Table S5. Relationship of the Nutrition Index-nutrient and Nutrition Index-lab/exam with frailty, using multivariable-adjusted ordinary least squares regression analyses. Table S6. Relationship of the Nutrition Index-nutrient and Nutrition Index-lab/exam with mortality, using multivariable-adjusted Cox regression analysis. Table S7. Coefficients and p values for the 3-way interactions of each dietary score with age and sex on frailty. Table S8. Coefficients and p values for the 4-way interactions of each dietary score with age, sex, and frailty on mortality. [file 12916_2021_1918_MOESM1_ESM.docx]

**Table S1. Dietary variables included in calculations of the dietary scores**

|  | **NI** | **E-DII** | **HEI-2015** | **MDS** | **DASH** |
| --- | --- | --- | --- | --- | --- |
| Energy^*^ | x | x | x |  | x |
| Protein | x | x |  |  | x |
| Carbohydrate | x | x |  |  |  |
| Total fatty acids |  | x |  |  | x |
| Saturated fatty acids | x | x | x | x | x |
| MUFAs |  | x | x | x |  |
| PUFAs |  | x | x |  |  |
| Omega-3 fatty acids |  | x |  |  |  |
| Omega-6 fatty acids |  | x |  |  |  |
| EPA and DHA | x |  |  |  |  |
| Cholesterol |  | x |  |  | x |
| Fiber |  | x |  |  | x |
| Vitamin A | x | x |  |  |  |
| Vitamin B1 | x | x |  |  |  |
| Vitamin B2 | x | x |  |  |  |
| Vitamin B3 | x | x |  |  |  |
| Vitamin B6 | x | x |  |  |  |
| Vitamin B12 |  | x |  |  |  |
| Folate | x | x |  |  |  |
| Vitamin C | x | x |  |  |  |
| Vitamin D |  | x |  |  |  |
| Vitamin E |  | x |  |  |  |
| Beta-carotene |  | x |  |  |  |
| Calcium |  |  |  |  | x |
| Copper | x |  |  |  |  |
| Iron |  | x |  |  |  |
| Magnesium |  | x |  |  | x |
| Phosphorus | x |  |  |  |  |
| Potassium |  |  |  |  | x |
| Selenium | x | x |  |  |  |
| Sodium | x |  | x |  | x |
| Zinc |  | x |  |  |  |
| Alcohol |  | x |  | x |  |
| Caffeine |  | x |  |  |  |
| Dairy product |  |  | x | x |  |
| Whole grains |  |  | x | x |  |
| Refined grains |  |  | x | x |  |
| Fruit juice |  |  | x |  |  |
| Whole fruits |  |  | x | x |  |
| Nuts |  |  |  | x |  |
| Total vegetables |  |  | x | x |  |
| Greens and beans |  |  | x |  |  |
| Red meat and product |  |  | x | x |  |
| Poultry |  |  | x | x |  |
| Seafood |  |  | x | x |  |
| Plant proteins |  |  | x |  |  |
| Added sugars |  |  | x |  |  |
| Nutrition-related blood tests | x |  |  |  |  |
| Anthropometric measurements | x |  |  |  |  |

DASH, Dietary approaches to stop hypertension; DHA, docosahexaenoic acid; E-DII, Energy-density Dietary inflammatory index; EPA, eicosapentaenoic acid; FI, Frailty index; HEI-2015, Healthy eating index-2015; MDS, Mediterranean diet score; MUFAs, Monounsaturated fatty acids; NI, Nutrition Index; PUFAs, Polyunsaturated fatty acids

*Energy was not included as an individual item in the E-DII, HEI-2015 and DASH but items within these scores, were adjusted for energy level.

**Table S2. 36-item Frailty Index**

| **Self-reported Frailty Index items** | |
| --- | --- |
| 1. Angina/angina pectoris | 14. Difficulty lifting or carrying |
| 2. Heart attack | 15. Difficulty walking between rooms on same floor |
| 3. Coronary heart disease | 16. Difficulty standing up from an armless chair |
| 4. Stroke | 17. Difficulty getting in and out of bed |
| 5. Thyroid condition | 18. Difficulty dressing yourself |
| 6. Cancer | 19. Difficulty grasping/holding small objects |
| 7. Arthritis | 20. Difficulty attending social events |
| 8. High blood pressure | 21. Self-reported health |
| 9. Diabetes mellitus | 22. Frequency of healthcare use |
| 10. Weak/failing kidneys | 23. Health compared to 1 year ago |
| 11. Confusion or inability to remember things | 24. Overnight hospital stays |
| 12. Difficulty managing money | 25. Medications |
| 13. Difficulty stooping, crouching, kneeling |  |
| **Laboratory Frailty Index items** | |
| 26. Pulse rate (60-99 bpm) | 32. Red cell distribution width (≤14.6%) |
| 27. Systolic blood pressure (90-140 mmHg) | 33. Lactate dehydrogenase (≤190 U/L) |
| 28. Pulse pressure (30-60 mmHg) | 34. Alkaline phosphatase (≤115 U/L) |
| 29. Platelet count SI (150-450 unit 1000 cells/uL) | 35. Uric acid (M: 240-510, F: 160-430 umol/L) |
| 30. Blood urea nitrogen (3-20 mg/dL) | 36. Total calcium (2.0-2.5 mmol/L) |
| 31. Bicarbonate (≤28 mmol/L) |  |

F = female; M = male

**Table S3. Association of participants’ baseline characteristics with frailty, using univariate linear regression analyses and with mortality risk, using Cox regression analysis**

|  | **Frailty** | | | **8-year mortality** | |
| --- | --- | --- | --- | --- | --- |
|  | **Unstandardized**  **Beta-coefficient (95%CI)** | **Standardized**  **Beta-coefficient** | ***p*-value** | **Hazard ratio (95%CI)** | ***p*-value** |
| Age (per 1 year) | 0.004 (0.004 to 0.004) | 0.56 | <0.001 | 1.09 (1.08,1.09) | <0.001 |
| Sex (female) | 0.012 (0.008 to 0.015) | 0.05 | <0.001 | 0.65 (0.58,0.74) | <0.001 |
| Education (ref. less than high school) |  |  |  |  |  |
| High school | -0.024 (-0.030 to -0.019) | -0.10 | <0.001 | 0.74 (0.64,0.86) | <0.001 |
| Some college/ associate education | -0.020 (-0.022 to -0.017) | -0.17 | <0.001 | 0.55 (0.48,0.64) | <0.001 |
| College graduate or more | -0.021 (-0.022 to -0.019) | -0.26 | <0.001 | 0.42 (0.35,0.50) | <0.001 |
| Marital status (ref. never married) |  |  |  |  |  |
| Married | 0.030 (0.026 to 0.035) | 0.12 | <0.001 | 1.94 (1.55,2.42) | <0.001 |
| Widowed | 0.075 (0.071 to 0.078) | 0.56 | <0.001 | 8.80 (6.95,11.13) | <0.001 |
| Divorced or separated | 0.020 (0.018 to 0.022) | 0.28 | <0.001 | 2.51 (1.94,3.24) | <0.001 |
| Full-time working | -0.105 (-0.109 to -0.102) | -0.46 | <0.001 | 0.17 (0.15,0.20) | <0.001 |
| Smoking status (ref. never) |  |  |  |  |  |
| Former | 0.048 (0.044 to 0.053) | 0.20 | <0.001 | 2.18 (1.92,2.48) | <0.001 |
| Current | 0.006 (0.004 to 0.008) | 0.05 | <0.001 | 1.13 (0.96,1.32) | 0.150 |
| Body mass index (kg/m^2^) (ref. 18.5-24.9) |  |  |  |  |  |
| <18.5 | 0.029 (0.016 to 0.042) | 0.07 | <0.001 | 1.69 (1.16,2.46) | 0.006 |
| 25.0-29.9 | 0.006 (0.004 to 0.008) | 0.06 | <0.001 | 1.04 (0.90,1.21) | 0.582 |
| ≥30 | 0.016 (0.014 to 0.017) | 0.20 | <0.001 | 0.87 (0.75,1.02) | 0.056 |
| Energy intake (per 100 kcal) | -0.002 (-0.002 to -0.002) | -0.19 | <0.001 | 0.96 (0.95,0.97) | <0.001 |

Kcal, kilocalories; kg, kilogram; m, meter; ref., reference

**Table S4. Correlation of the Nutrition Index-nutrient and Nutrition Index-lab/exam with other dietary scores**

|  | | **Correlation coefficient (*r*)** | ***p*-value** |
| --- | --- | --- | --- |
| NI-nutrient* | NI | 0.93 | <0.001 |
|  | NI-lab/exam | 0.16 | <0.001 |
|  | E-DII | 0.16 | <0.001 |
|  | HEI-2015 | -0.20 | <0.001 |
|  | MDS | -0.10 | <0.001 |
|  | DASH | 0.02 | <0.001 |
| NI-lab/exam* | NI | 0.53 | <0.001 |
|  | E-DII | 0.05 | <0.001 |
|  | HEI-2015 | -0.09 | <0.001 |
|  | MDS | -0.06 | <0.001 |
|  | DASH | -0.02 | <0.001 |

DASH, Dietary approaches to stop hypertension; E-DII, Energy-density Dietary inflammatory index; HEI-2015, Healthy eating index-2015; MDS, Mediterranean diet score; NI, Nutrition Index.

Higher NI and E-DII score and lower HEI-2015, MDS, and DASH scores represents worse dietary pattern/intake.

*Here we separated the 31-item Nutrition Index into two indices: the NI-nutrient which included only the 18 nutrients and the NI-lab/exam which included 10 nutrition-related blood tests and 3 anthropometric measurements.

**Table S5. Relationship of the Nutrition Index-nutrient and Nutrition Index-lab/exam with frailty, using multivariable-adjusted ordinary least squares regression analyses**

| **Dietary scores** | **Unstandardized**  **Beta-coefficient (95%CI)** | **Standardized**  **Beta-coefficient** | ***p*-value** |
| --- | --- | --- | --- |
| NI-nutrient (per 0.1 point) | 0.002 (0.001 to 0.003) | 0.04 | <0.001 |
| NI-lab/exam (per 0.1 point)* | -0.002 (-0.006 to 0.001) | -0.03 | 0.121 |
| NI-lab/exam squared | 0.003 (0.003 to 0.004) | 0.26 | <0.001 |
| E-DII (per 1 point) | 0.001 (0.000 to 0.002) | 0.02 | 0.003 |
| NI-nutrient (per 0.1 point) | 0.002 (0.001 to 0.003) | 0.04 | <0.001 |
| HEI-2015 (per 10 points) | 0.004 (-0.002 to 0.011) | 0.05 | 0.168 |
| HEI-2015 squared | -0.001 (-0.001 to 0.000) | -0.09 | 0.014 |
| NI-nutrient (per 0.1 point) | 0.002 (0.001 to 0.002) | 0.04 | <0.001 |
| MDS (per 1 point) | -0.003 (-0.004 to -0.002) | -0.04 | <0.001 |
| NI-nutrient (per 0.1 point) | 0.002 (0.001 to 0.003) | 0.04 | <0.001 |
| DASH (per 1 point) | -0.001 (-0.002 to -0.001) | -0.02 | 0.002 |
| NI-nutrient (per 0.1 point) | 0.002 (0.001 to 0.003) | 0.05 | <0.001 |
| E-DII (per 1 point) | 0.001 (0.000 to 0.002) | 0.02 | 0.005 |
| NI-lab/exam (per 0.1 point) | 0.017 (0.016 to 0.018) | 0.23 | <0.001 |
| HEI-2015 (per 10 points) | -0.003 (-0.004 to -0.002) | -0.04 | <0.001 |
| NI-lab/exam (per 0.1 point) | 0.017 (0.016 to 0.018) | 0.23 | <0.001 |
| MDS (per 1 point) | -0.003 (-0.004 to -0.002) | -0.04 | <0.001 |
| NI-lab/exam (per 0.1 point) | 0.017 (0.016 to 0.018) | 0.23 | <0.001 |
| DASH (per 1 point) | -0.001 (-0.002 to -0.001) | -0.02 | 0.001 |
| NI-lab/exam (per 0.1 point) | 0.017 (0.016 to 0.018) | 0.23 | <0.001 |
| E-DII (per 1 point) | 0.001 (0.000 to 0.002) | 0.01 | 0.058 |
| NI-nutrient (per 0.1 point) | 0.001 (0.001 to 0.002) | 0.03 | <0.001 |
| NI-lab/exam (per 0.1 point) | 0.017 (0.016 to 0.018) | 0.23 | <0.001 |
| HEI-2015 (per 10 points) | -0.002 (-0.004 to -0.001) | -0.03 | <0.001 |
| NI-nutrient (per 0.1 point) | 0.001 (0.000 to 0.002) | 0.02 | <0.001 |
| NI-lab/exam (per 0.1 point) | 0.017 (0.016 to 0.018) | 0.22 | <0.001 |
| MDS (per 1 point) | -0.003 (-0.003 to -0.002) | -0.04 | <0.001 |
| NI-nutrient (per 0.1 point) | 0.001 (0.001 to 0.002) | 0.03 | <0.001 |
| NI-lab/exam (per 0.1 point) | 0.017 (0.016 to 0.018) | 0.23 | <0.001 |
| DASH (per 1 point) | -0.001 (-0.002 to -0.001) | -0.02 | 0.010 |
| NI-nutrient (per 0.1 point) | 0.001 (0.001 to 0.002) | 0.03 | <0.001 |
| NI-lab/exam (per 0.1 point) | 0.017 (0.016 to 0.018) | 0.23 | <0.001 |

BMI, body mass index; DASH, Dietary approaches to stop hypertension; E-DII, Energy-density Dietary inflammatory index; FI, Frailty index; HEI-2015, Healthy eating index-2015; MDS, Mediterranean diet score; NI, Nutrition Index

All regression models were adjusted for age, sex, race, educational level, marital status, employment status, smoking status, study cohort, and BMI. Here we separated the 31-item nutrition Index into two indices: the NI-nutrient which included only the 18 nutrients, and the NI-lab/exam which included 10 nutrition-related blood tests and 3 anthropometric measurements.

Higher NI and E-DII score and lower HEI-2015, MDS, and DASH scores represents worse dietary pattern/intake.

In an initial model we tested the linear relationship, in the second model we added the squared term, and in the third model, we added the cubic term. We present results only for the highest order model that was statistically significant. If none of the models were statistically significant, we present the linear model. *This regression model was additionally adjusted for energy intake.

**Table S6. Relationship of the Nutrition Index-nutrient and Nutrition Index-lab/exam with mortality, using multivariable-adjusted Cox regression analysis**

| **Dietary scores** | **3-year mortality** | | **8-year mortality** | |
| --- | --- | --- | --- | --- |
|  | **Hazard ratio (95%CI)** | ***p*-value** | **Hazard ratio (95%CI)** | ***p*-value** |
| Adjusted for age, sex, race, educational level, marital status, employment status, smoking, study cohort, and BMI (basic covariates) | | | | |
| NI-nutrient (per 0.1 point) | 1.03 (0.99,1.07) | 0.120 | 1.03 (1.01,1.06) | 0.010 |
| NI-lab/exam (per 0.1 point) | 1.39 (1.30,1.49) | <0.001 | 1.35 (1.29,1.42) | <0.001 |
| Adjusted for basic covariates, and FI | | | | |
| NI-nutrient (per 0.1 point) | 1.06 (1.00,1.11) | 0.037 | 1.03 (1.00,1.05) | 0.031 |
| NI-lab/exam (per 0.1 point) | 1.24 (1.16,1.33) | <0.001 | 1.23 (1.17,1.29) | <0.001 |
| E-DII (per 1 point) | 1.02 (0.97,1.07) | 0.526 | 1.03 (0.99,1.07) | 0.074 |
| E-DII squared | 0.98 (0.96,1.00) | 0.073 | 0.99 (0.97,0.99) | 0.046 |
| NI-nutrient (per 0.1 point) | 1.03 (0.99,1.07) | 0.194 | 1.02 (0.99,1.05) | 0.111 |
| HEI-2015 (per 10 points) | 0.93 (0.87,1.00) | 0.064 | 0.95 (0.90,0.99) | 0.007 |
| NI-nutrient (per 0.1 point) | 1.02 (0.98,1.06) | 0.319 | 1.02 (0.99,1.05) | 0.176 |
| MDS (per 1 point) | 0.94 (0.89,1.00) | 0.069 | 0.94 (0.90,0.98) | 0.002 |
| NI-nutrient (per 0.1 point) | 1.03 (0.99,1.07) | 0.186 | 1.04 (1.01,1.08) | 0.018 |
| DASH (per 1 point) | 0.96 (0.91,1.02) | 0.148 | 0.96 (0.93,0.99) | 0.050 |
| NI-nutrient (per 0.1 point) | 1.03 (0.99,1.07) | 0.134 | 1.03 (1.01,1.05) | 0.039 |
| E-DII (per 1 point) | 1.03 (0.98,1.08) | 0.254 | 1.04 (1.01,1.07) | 0.016 |
| NI-lab/exam (per 0.1 point) | 1.24 (1.15,1.33) | <0.001 | 1.20 (1.14,1.26) | <0.001 |
| HEI-2015 (per 10 points) | 0.93 (0.87,0.99) | 0.045 | 0.94 (0.90,0.98) | 0.005 |
| NI-lab/exam (per 0.1 point) | 1.24 (1.15,1.33) | <0.001 | 1.20 (1.14,1.26) | <0.001 |
| MDS (per 1 point) | 0.95 (0.89,1.01) | 0.090 | 0.94 (0.90,0.98) | 0.003 |
| NI-lab/exam (per 0.1 point) | 1.24 (1.15,1.33) | <0.001 | 1.20 (1.14,1.26) | <0.001 |
| DASH (per 1 point) | 0.96 (0.91,1.02) | 0.178 | 0.97 (0.93,1.00) | 0.066 |
| NI-lab/exam (per 0.1 point) | 1.24 (1.15,1.33) | <0.001 | 1.20 (1.15,1.26) | <0.001 |
| E-DII (per 1 point) | 1.02 (0.97,1.08) | 0.376 | 1.03 (1.00,1.07) | 0.041 |
| NI-nutrient (per 0.1 point) | 1.02 (0.98,1.06) | 0.330 | 1.02 (0.99,1.04) | 0.205 |
| NI-lab/exam (per 0.1 point) | 1.23 (1.15,1.33) | <0.001 | 1.20 (1.14,1.25) | <0.001 |
| HEI-2015 (per 10 points) | 0.99 (0.99,1.00) | 0.081 | 0.94 (0.90,0.99) | 0.016 |
| NI-nutrient (per 0.1 point) | 1.02 (0.98,1.06) | 0.472 | 1.02 (0.99,1.04) | 0.262 |
| NI-lab/exam (per 0.1 point) | 1.23 (1.15,1.33) | <0.001 | 1.20 (1.14,1.25) | <0.001 |
| MDS (per 1 point) | 0.95 (0.89,1.01) | 0.116 | 0.94 (0.91,0.98) | 0.005 |
| NI-nutrient (per 0.1 point) | 1.02 (0.98,1.06) | 0.301 | 1.02 (0.99,1.05) | 0.133 |
| NI-lab/exam (per 0.1 point) | 1.23 (1.15,1.33) | <0.001 | 1.20 (1.14,1.25) | <0.001 |
| DASH (per 1 point) | 0.96 (0.91,1.02) | 0.190 | 0.97 (0.93,1.00) | 0.077 |
| NI-nutrient (per 0.1 point) | 1.02 (0.98,1.06) | 0.242 | 1.02 (0.99,1.05) | 0.082 |
| NI-lab/exam (per 0.1 point) | 1.23 (1.15,1.33) | <0.001 | 1.20 (1.14,1.25) | <0.001 |

BMI, body mass index; DASH, Dietary approaches to stop hypertension; E-DII, Energy-density Dietary inflammatory index; FI, Frailty index; HEI-2015, Healthy eating index-2015; MDS, Mediterranean diet score; NI, Nutrition Index

2007-2012 NHANES cohorts included in the analysis and mortality was identified up to December 2015. We separated the 31-item nutrition Index into two indices: the NI-nutrient which included only the 18 nutrients and the NI-lab/exam which included 10 nutrition-related blood tests and 3 anthropometric measurements.

Higher NI and E-DII score and lower HEI-2015, MDS, and DASH scores represents worse dietary pattern/intake.

In an initial model we tested the linear relationship, in the second model we added the squared term, and in the third model, we added the cubic term. We present results only for the highest order model that was statistically significant. If none of the models were statistically significant, we present the linear model.

**Table S7.** **Coefficients and *p* values for the 3-way interactions of each dietary score with age and sex on frailty.**

| **Dietary scores** | **Beta-coefficients** | ***p*-value for interaction** |
| --- | --- | --- |
| NI | 0.63 | 0.667 |
| E-DII | -0.04 | 0.509 |
| HEI-2015 | 0.02 | 0.908 |
| MDS | -0.04 | 0.681 |
| DASH | 0.06 | 0.491 |
| NI-nutrient | 0.21 | 0.859 |
| NI-lab/exam | 0.33 | 0.238 |

DASH, Dietary approaches to stop hypertension; E-DII, Energy-density Dietary inflammatory index; HEI-2015, Healthy eating index-2015; MDS, Mediterranean diet score; NI, Nutrition Index. Here we separated the 31-item nutrition Index into two indices: the NI-nutrient which included only the 18 nutrients and the NI-lab/exam which included 10 nutrition-related blood tests and 3 anthropometric measurements.

**Table S8. Coefficients and *p* values for the 4-way interactions of each dietary score with age, sex, and frailty on mortality.**

| **Dietary scores** | **3-year mortality** | | **8-year mortality** | |
| --- | --- | --- | --- | --- |
|  | **Beta-coefficients** | ***p* for interaction** | **Beta-coefficients** | ***p* for interaction** |
| NI | 0.00 | 0.244 | 0.00 | 0.159 |
| E-DII | 0.00 | 0.356 | 0.00 | 0.292 |
| HEI-2015 | 0.00 | 0.296 | 0.00 | 0.142 |
| MDS | 0.00 | 0.267 | 0.00 | 0.061 |
| DASH | 0.00 | 0.100 | 0.00 | 0.160 |
| NI-nutrient | 0.00 | 0.893 | 0.00 | 0.990 |
| NI-lab/exam | 0.00 | 0.310 | 0.00 | 0.340 |

DASH, Dietary approaches to stop hypertension; E-DII, Energy-density Dietary inflammatory index; HEI-2015, Healthy eating index-2015; MDS, Mediterranean diet score; NI, Nutrition Index. Here we separated the 31-item nutrition Index into two indices: the NI-nutrient which included only the 18 nutrients and the NI-lab/exam which included 10 nutrition-related blood tests and 3 anthropometric measurements.
